# Insufflation with Humidified and Heated Carbon Dioxide in Short-Term Laparoscopy: A Double-Blinded Randomized Controlled Trial

**DOI:** 10.1155/2015/412618

**Published:** 2015-01-28

**Authors:** Anja Herrmann, Rudy Leon De Wilde

**Affiliations:** Clinic of Gynecology, Obstetrics and Gynecological Oncology, University Hospital for Gynecology, Pius-Hospital Oldenburg, School of Medicine and Health Sciences, Carl von Ossietzky University of Oldenburg, 26121 Oldenburg, Germany

## Abstract

*Background.* We tested the hypothesis that warm-humidified carbon dioxide (CO_2_) insufflation would reduce postoperative pain and morphine requirement compared to cold-dry CO_2_ insufflation. *Methods.* A double-blinded, randomized, controlled trial was conducted to compare warm, humidified CO_2_ and cold-dry CO_2_. Patients with benign uterine diseases were randomized to either treatment (*n* = 48) or control (*n* = 49) group during laparoscopically assisted vaginal hysterectomy. Primary endpoints of the study were rest pain, movement pain, shoulder-tip pain, and cough pain at 2, 4, 6, 24, and 48 hours postoperatively, measured by visual analogue scale. Secondary outcomes were morphine consumption, rejected boli, temperature change, recovery room stay, and length of hospital stay. *Results.* There were no significant differences in all baseline characteristics. Shoulder-tip pain at 6 h postoperatively was significantly reduced in the intervention group. Pain at rest, movement pain, and cough pain did not differ. Total morphine consumption and rejected boli at 24 h postoperatively were significantly higher in the control group. Temperature change, recovery room stay, and length of hospital were similar. *Conclusions.* Warm, humidified insufflation gas significantly reduces postoperative shoulder-tip pain as well as morphine demand. This trial is registered with *Clinical Trial Registration Number* DRKS00003853 (German Clinical Trials Register (DRKS)).

## 1. Introduction

During laparoscopic interventions, dry carbon dioxide (CO_2_) gas at room temperature is used to create and maintain pneumoperitoneum. This stands in contrast to the physiological conditions of the peritoneal cavity at body temperature of 37°C and continuous moistening of the peritoneum by the peritoneal fluid [1]. Studies in both animal models and clinical trials in humans have demonstrated that insufflated CO_2_ has adverse effects on the peritoneal cells [2]. Desiccation and damage of the peritoneum occur, along with the alteration of physiological processes and enhanced release of inflammation-promoting mediators [2], potentially leading to an increased sensation of pain following surgery [3]. It is a presently accepted paradigm that patients who are fast-tracked for early postoperative release (where feasible) in ERAS (enhanced recovery after surgery) programmes will be an additional cost to the health system if early postoperative pain levels are not adequately controlled.

Recently, over the previous several years, various studies have been conducted to investigate the impact of humidified and heated insufflation gas on the intensity of postoperative pain as perceived by patients as well as its impact on body temperature. Invariably, the available studies have differing study designs, statistical power, and clinical outcomes, introducing uncertainty in decision-making pathways to decide whether or not a laparoscopic humidification system should be implemented in the clinical routine. To evaluate this in our department, we conducted a study on laparoscopic-assisted vaginal hysterectomy (LAVH). This procedure was chosen as it is frequently performed, conducted in a predefined order, and can therefore be standardized. Thus, unlike many other studies in the field of gynecology, in the present study potential impacts on the results caused by different surgical procedures are largely avoided. The primary aim of this study was to determine the impact of a laparoscopic gas conditioning system on postoperative pain development following LAVH.

## 2. Materials and Methods

This was a single-center, double-blind, parallel-group, randomized controlled trial conducted in the Department of Gynecology, Obstetrics, and Gynecological Oncology, University Clinic for Gynecology, Pius-Hospital, Carl von Ossietzky University, Oldenburg, Germany, between April 2012 and January 2013. The trial was conducted to compare the use of warm, humidified insufflation CO_2_ gas and cold, nonhumidified CO_2_ insufflation gas (standard gas) for several intra- and postoperative outcomes in LAVH.

### 2.1. Endpoints of the Study

The primary endpoint of the study was postoperative pain development at 2, 4, 6, 24, and 48 hours postoperatively for rest pain, movement pain, shoulder-tip pain, and cough pain as measured by visual analogue scale (VAS). Secondary endpoints were morphine consumption, rejected boli, temperature change during surgery, length of time spent in the recovery room, and duration of inpatient stay.

### 2.2. Eligibility Criteria

Eligible patients were aged 18 years and over with benign uterine condition, at least one vaginal delivery, no previous surgeries in the case history that indicated extensive cicatrisation, no longitudinal laparotomy in the case history, no current oncological disease, no concurrent chronic disease requiring continuous intake of analgesics, ability to understand the study procedure, sonographic estimation of uterus weight below 400 grams, and preoperative estimation of surgery time between 1 and 2 hours. It was assumed that patients who have never given birth vaginally could experience more pain postoperatively due to a tighter vagina which is often associated with more manipulation during surgery to remove the uterus vaginally. Therefore, these patients were at first excluded due to possible effect confounding, but after 3 1/2 months of recruitment (16 patients, one “drop-out”) it was recognized that these patients represent a substantive proportion of the patient population as many women had only birthed by cesarean section. To increase the recruitment rate and complete the study within a realistic time frame, it was decided to include patients who did not give vaginal birth. The generalizability of the results to the patient population in our hospital was thus extended.

### 2.3. Ethic Committee and Trial Registration

The study was approved by the Ethics Committee of the University of Göttingen, Germany, in April 2012 (committee reference number 16/2/12). The study was conducted according to the Good Clinical Practice guidelines provided by International Conference on Harmonisation (ICH). The study was conducted with the understanding and the consent of the patients. The trial was prospectively registered online with the German Clinical Trials Register (DRKS) which is an official recognized Primary Registry in the WHO Registry Network (unique DRKS study number DRKS00003853).

### 2.4. Study Course

The admission plan called for daily scrutinization of eligible patients and after an initial physical examination by a senior physician a decision was made as to whether the patient would be requested to participate in the study. All patients gave their informed consent one day before surgery and prior to any study activity. All patients received standard induced anesthesia with propofol, esmeron, and fentanyl (0.25 *μ*g) and maintenance of anesthesia with propofol titrated to effect. As the study was double-blinded, disclosure of treatment group was effected while patients were under anesthesia by a sealed, opaque, sequentially numbered envelope stating the treatment allocation. The study nurse responsible for pain measurement was excluded from any activities in the OR as well as from relevant staff and no further information was communicated to the study nurse. According to randomization, pneumoperitoneum was then either established and maintained with either cold (room temperature), dry (0% humidity) CO_2_ or with warm (35 ± 2°C), humidified (98% humidity) CO_2_. Humidification of CO_2_ was by the HumiGard MR 860 Surgical Humidification System (Fisher & Paykel Healthcare Limited, Auckland, New Zealand) [4, 5]. For CO_2_ insufflation, THERMOFLATOR from KARL STORZ was used (KARL STORZ GmbH & Co. KG, Tuttlingen, Germany) with pressure set to 14 mmHg and an upper gas flow limit of 6.5 L/min. All procedures were performed by several participating senior physicians. Study participants received LAVH with or without bilateral or unilateral salpingo-oophorectomy (SOO). The operation performed in the hospital can be broadly divided into three parts. The first part was a conventional laparoscopy in which the fallopian tube was dissected from the uterus (LAVH without SOO) or in which the infundibulopelvic ligament was dissected from the pelvic wall (LAVH with SOO) to prepare the uterus for vaginal removal. For abdominal entry during laparoscopy three incisions were made: one 10-millimeter incision in the umbilicus for the camera and two 5-millimeter incisions on the left and right lower abdomen for the instruments [6]. The second part of the operation was the vaginal removal of the uterus as described by Goolab [7]. During the third part pneumoperitoneum was again established and laparoscopy was performed again to remove potentially pooled blood in the rectouterine excavation as well as to terminate any existing bleeding of the vaginal cuff suture. The surgery site was examined for bleeding, and abdominal incisions were closed by interrupted sutures and sterile dressing was applied. A cystoscopy was performed and a permanent transurethral catheter subsequently introduced and retained along with a vaginal tamponade for 24 hours. Under optimal conditions the total laparoscopic time is about 20 min as the first part is completed in approximately 15 min with the last part taking approximately 5 min to complete. If pathologies are present this time course could be extended. Therefore, this operation can be regarded as short-term laparoscopy. Between the two laparoscopies, the pneumoperitoneum is deflated in order to prevent gas escaping into the atmosphere through the vagina. Therefore pneumoperitoneum must be reestablished for the second laparoscopic part.

### 2.5. Outcome Measures

Surgery duration was recorded by the OR staff (defined as time between first trocar insertion and last suture of the trocar incision), as well as the length of time the pneumoperitoneum was maintained for the first and second laparoscopic part (period between gas insufflation and gas exsufflation). Volume of insufflated CO_2_ was recorded. Body temperature was recorded by intranasal probe at the beginning and the end of procedure by the anesthetic staff. Theater ambient (room) temperature was preoperatively adjusted to 18°C and recorded continuously by electronic thermometer from where the temperature at the mid-point of the operation was electronically obtained. All patients received an upper body thermal blanket both intra- and postoperatively (Warm Touch, Covidien, Mansfield, USA). Applied intraoperative fluids were preheated to 38°C. After surgery, patients were transferred to the recovery room, and after evaluation of pain levels, postoperative nausea, and observation by nursing staff they were transferred to the ward. A study nurse, blinded to treatment allocation, recorded patients' pain levels by visual analogue score (VAS) at 2, 4, 6, 24, and 48 hours postoperatively in the following sequence: pain at rest, movement pain, shoulder-tip pain, and cough pain. As patients are usually not able to stand on the operating day, they were asked to rotate themselves, in bed, to the right and to the left side. At 24 and 48 hours postoperatively (first and second postoperative day), patients were asked about their pain at rest prior to arising in the morning, with all other pain levels recorded after ablutions. Shoulder-tip pain was defined as any pain that could be associated with gas insufflation, for example, pressing pain under the costal arch, chest pain during breathing or movement, and shoulder-tip pain in the proper sense. Pain was not regarded as gas related if the pain could be due to orthopedic problems. For measurement of cough pain, patients were asked to cough, strongly, twice. All pain scores were recorded by a single individual.

### 2.6. Analgesic Consumption

All patients received pain medication according to a standardized protocol, as well as metamizole (1 gram i.v.) at the end of anesthesia. In the recovery room, patients received morphine titrated to analgesia or VAS score 3, respectively, followed by connection of the PCA (patient controlled analgesia) pump with the following settings: 1.5 mg morphine per bolus, bolus lock for 10 minutes, and a maximum of 30 mg morphine per 4 hours. Clonidine was not allowed due to its analgesic effect. Patients were allowed metamizole 1 gram i.v. on demand, or if they were allergic to metamizole; then 1-gram acetaminophen was administered. In the ward, patients received 1-gram metamizole i.v. in the evening on the operating day, and from the first postoperative day until the third postoperative day 1-gram metamizole (alternatively acetaminophen 1 gram) was administered four times a day at 6 a.m., noon, 6 p.m., and midnight. Additionally, patients were allowed their PCA pumps up to the second postoperative day for additional pain reduction when necessary. PCA pumps were software-monitored in order to obtain the applied boli data, which included boli dispensing requests which fell within the 10 min lockout period. Morphine consumption on the operating day referred to the time span between connection of the PCA pump and midnight; morphine consumption 24 hours postoperatively refers to the time span between 0:00 hours and 24:00 hours on the first postoperative day and morphine consumption at 48 hours refers to the time span between 0:00 hours and disconnection of the PCA pump on the second postoperative day. The time from connection of the PCA pump until midnight of the operation day was measured to ensure that the time patients had to use the pump on the operation day was equally distributed between groups. Intraoperative morphine and morphine applied in recovery room before connection of the PCA-pump were measured to ensure that patients in each group started with the same dose of morphine before they were able to self-medicate by PCA.

### 2.7. Statistics

Patients were randomly assigned to one of two parallel groups in 1 : 1 ratio to receive either warm, humidified or cold, nonhumidified gas. Randomization was effected with RITA (Randomization in Treatment Arms) software, version 1.27. Permuted-block randomization with block length of 4 and 6 was used. The sample size has been determined statistically by means of a power analysis. The primary endpoint was the investigation of postoperative pain by means of a VAS with values between 0 and 10. Normal distribution with the same standard deviation was assumed in both groups. The standard deviation used to calculate the power was obtained from previous studies and fixed at 2.5 [8], and difference of 1.5 points on the VAS was considered to be clinically significant. Significance was accepted at *p* ≤ 0.05 and the probability of type-II errors (*β*) at 20%. This yielded a sample size of 44 subjects per treatment group at a power of 1-*β* = 80%. A repeated-measures ANOVA was fitted to within-subjects condition (i.e., time), with Greenhouse-Geiser's epsilon correction to degrees of freedom where deviations from sphericity were significant. Fisher's least significant difference (LSD) was used to evaluate significance in longitudinal data. Between-groups differences are presented as mean (95% CI) or median (95% CI) for parametric and nonparametric data, respectively, using the Mann-Whitney* U* test for nonparametric continuous variables with* t*-tests for continuous variables. The two treatment conditions were “Control” which received standard of care which was cold, dry CO_2_ for insufflation, and “Treatment” which received warm humidified CO_2_. Significance tests always report the two-sided value but also the upper one-sided value where it was significant, as the two-sided value gives the probability of different distributions for the two treatments, while the upper one-sided value tests if control (*x*) has more pain, analgesic consumption or rejected boli than treatment (*y*). Data were analyzed in Genstat V.16 (VSN International Ltd; http://www.vsni.co.uk), and statistical significance was accepted at the 0.05 level with no subgroup analyses planned or undertaken. There were also no interim analyses.

### 2.8. Allocation Concealment and Blinding

Based on the randomization list generated with RITA, an independent, external person prepared sequentially numbered, opaque, sealed envelopes. Envelopes were labelled with the respective randomization list number (subjects 1, 2, 3, etc.). The envelope contained the allocation to the respective treatment group printed on opaque paper in light letters, which served as additional privacy screen. After informed consent, patients received a study number identical to the number on the envelope. If a patient was a “drop-out” the corresponding envelope was discarded and the next patient received the next envelope. Envelopes were stored in a locked room and handed to OR staff by the study nurse shortly before surgery and after induction of anesthesia.

The recording of pain, which was double-blinded with regard to the type of the treatment given, was carried out by a study nurse. All persons involved in the study were expressly advised that neither the patients nor the study nurse may obtain knowledge of the type of the treatment given. Patients were informed of the blinding via the patient information sheet and within the scope of the patient briefing.

## 3. Results

**Figure 1:**
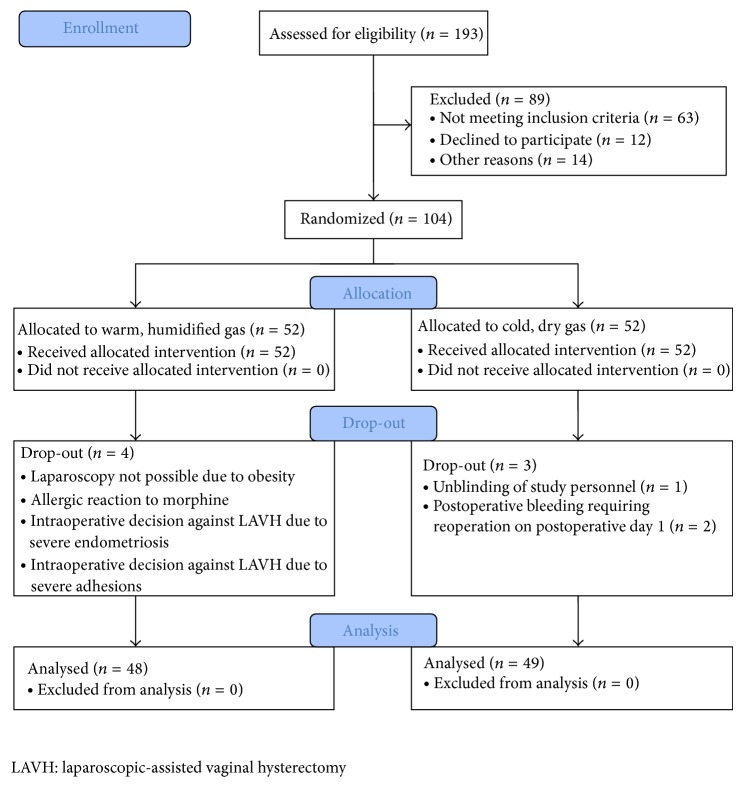
CONSORT flow diagram: patient inclusion.

**Figure 2:**
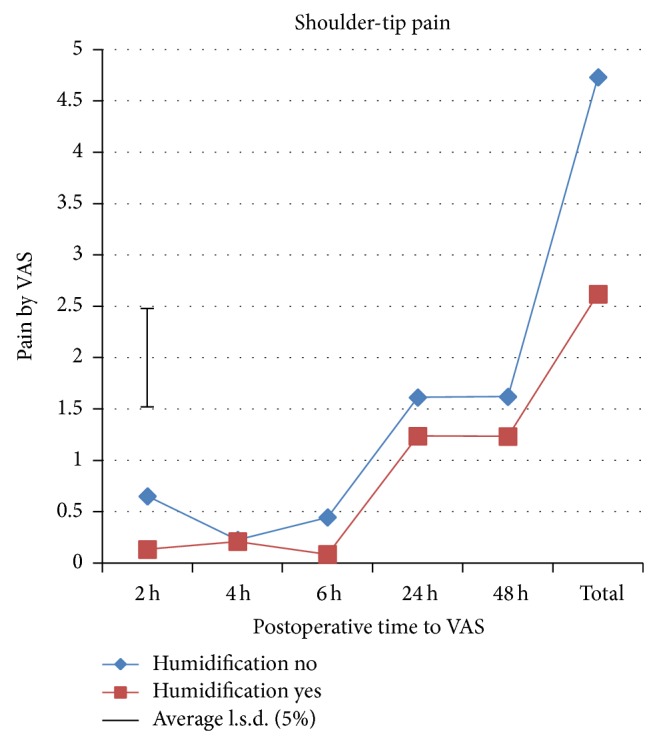
Shoulder-tip pain assessed by VAS.

**Figure 3:**
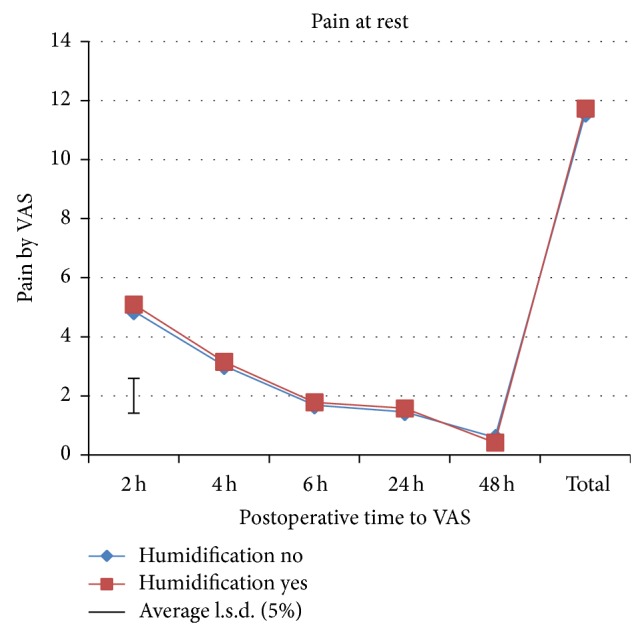
Rest pain assessed by VAS.

**Figure 4 fig4:**
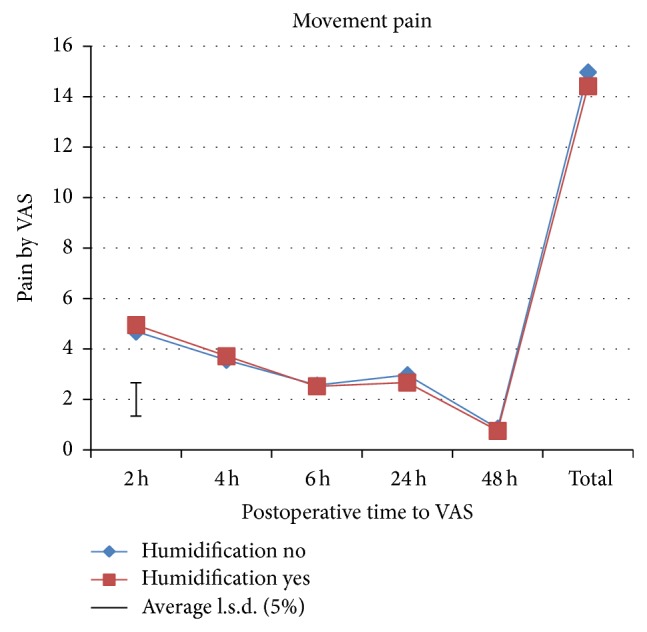
Movement pain assessed by VAS.

**Figure 5:**
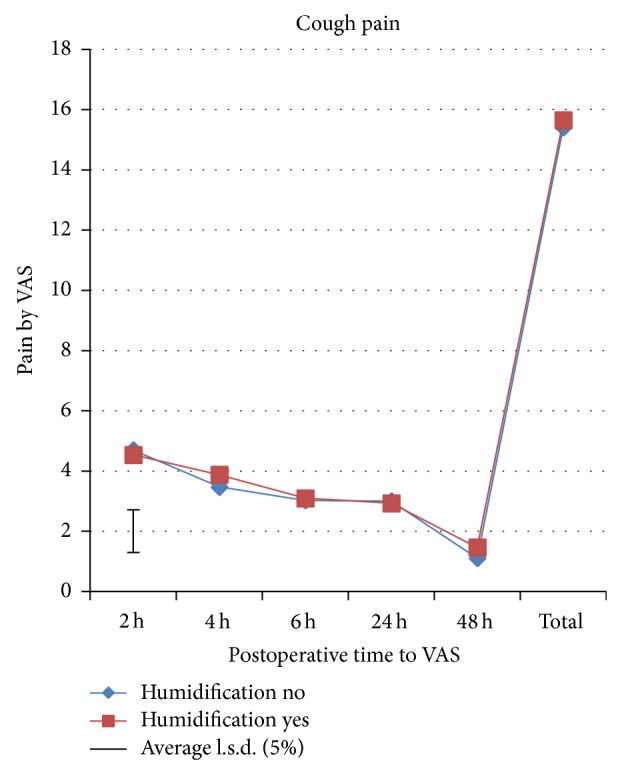
Cough pain assessed by VAS.

A total of 193 patients were preoperatively assessed for eligibility, of which 89 patients were excluded from the study as they did not meet the inclusion criteria mainly due to additional procedures like cystocele and/or rectocele repair, or they declined to participate or due to other reasons. A total of 104 patients were randomized to one of the two study groups. All patients received the allocated intervention. Four patients in the intervention group and 3 patients in the control group were excluded from the study after randomization (Figure 1). Therefore, 48 patients in the intervention group and 49 patients in the control group were available for analysis. Baseline characteristics are shown in Table 1. There were no significant differences between groups in all recorded baseline characteristics. Significant differences were found in shoulder-tip pain, morphine consumption, and rejected boli (Table 2). Divergence in shoulder-tip pain between treatments occurred after 4 h and reached significance at 6 h postoperatively (Figure 2). Shoulder-tip pain increased significantly in both treatment groups over time (*p* < 0.001), and this is in contrast to the other types of pain where the pain decreased significantly over time (*p* < 0.001) (Figures 3 [Fig fig4]–5). The control group experienced, on average, higher shoulder-tip pain than the humidified group at all times except at 4 h postoperatively. However, 23 patients (50%) in the intervention group did not experience shoulder-tip pain at any time during the study, in comparison with 16 patients (34%) in the control group. In patients who did experience shoulder-tip pain, the pain increased substantively after 6 h but stabilized at a higher plateau between 24 and 48 h. A significant difference in total shoulder-tip pain can be shown with the upper side *p* value. Total morphine consumption was significantly higher in the control group demonstrated by both the upper side and two-sided *p* value. This is consistent with morphine consumption in specific time points, which was significantly higher in the control group at both operation day and 24 h postoperatively although only the upper side *p* value showed significance. This is also reflected by the significant difference in the rejected boli 24 h postoperatively as it indicates that the patients would have used the PCA pump more often to reduce the pain if it had been allowed. Pain at rest, movement pain, and cough pain did not differ between both groups (Figures 3–5). Temperature change during operation, length of time in recovery room, and inpatient stay in hospital did also not differ between treatment groups (Table 2). There were 2 postoperative bleedings in the control group requiring reoperation on the first postoperative day. One patient in the intervention group had an allergic reaction to morphine. No other complications or adverse events occurred. No unblinding of any participants at any point during the conduct of the study took place.

## 4. Discussion

### 4.1. Brief Synopsis of the Key Findings

The current study revealed that using warm, humidified CO_2_ during insufflation compared to cold, nonhumidified insufflation gas significantly reduces total shoulder-tip pain, morphine consumption on operation day, and morphine consumption at 24 h postoperatively. Shoulder-tip pain in the treatment group was also significantly lower in the 6 h postoperative time period. Pain at rest, movement pain, and cough pain did not differ between groups. Total morphine consumption and rejected boli at 24 h postoperatively were also significantly higher in the control group. Temperature change during operation, length of time in recovery room, and inpatient stay in hospital did not differ between groups.

### 4.2. Consideration of Possible Mechanisms and Explanations

The fact that there were no observed differences between pain scores assessed by VAS (except for shoulder-tip pain) and that morphine use was statistically different between the groups suggests firstly that the self-administered morphine was effective at ameliorating the majority of the pain experienced by patients except for shoulder-tip pain and secondly that the self-administered morphine was an effect-confounder in the evaluation of the true difference in pain experienced between treatment groups. Of all the different kinds of pain analysed, shoulder-tip pain was the least responsive to morphine analgesic use, illustrating the severity of the pain. Morphine was therefore not as effective to control shoulder pain, as it was to control all other types of pain, such as rest pain. The fact that there is no difference in the pain between groups is evidence that the morphine controls the pain, but that the humidified group used less morphine to achieve the same pain control as the control group. It can therefore be assumed that the true pain felt by patients was reflected by the significant difference in morphine administered. However, it cannot be stated with absolute certainty whether the difference in morphine consumption is a surrogate parameter for the lower overall pain levels felt by the patients in the intervention group or the higher shoulder-tip pain experienced by patients in the control group. Statistically, it is unlikely that the effect occurred by chance, as shown by the relevant *p* values.

Several explanatory approaches for the development of shoulder-tip pain (STP) after laparoscopic surgery are current. It is assumed that the underlying process of STP is an irritation of phrenic nerve branches [9] caused by mechanical stretching of the diaphragm [10], injury to the crura of the diaphragm [11], or metabolic irritation [12]. A previous study by Jackson et al. (1996) also reported a significant relationship between STP and residual gas measured by X-ray imaging [13]. It is therefore likely that a multitude of factors are responsible for STP development after laparoscopy, as studies with different approaches have demonstrated a significant decrease of STP in their intervention group. In some studies the pain was reduced by using lower pressure for maintenance of pneumoperitoneum [10, 14, 15]. Other investigators reduced STP by administering analgesics into the abdomen [16–18] or by using a pulmonary recruitment maneuver to eliminate residual gas [19, 20]. The use of saline washout had no effect in one study [21], whereas another study demonstrated a reduction of the intensity but not the frequency of STP [15]. Similar studies have also compared heating of the insufflation gas without humidification, but this has been shown to further promote desiccation of the peritoneum and increase STP [22]. In our study, warming and humidification of the insufflation gas significantly reduced shoulder-tip pain. Histological studies have shown that carbon dioxide pneumoperitoneum is associated with a destruction of the peritoneal layer as well as with an alteration of the peritoneal cell metabolism due to desiccation and pressure leading to peritoneal acidosis and release of inflammatory mediators with a following inflammatory response [23–25]. Several studies therefore suggest that destruction of the peritoneal integrity contributes to the development of shoulder-tip pain. In an animal study, the application of warm, humidified gas led to faster dissipation of residual gas associated with a reduced duration of inflammation [26]. However, as shoulder-tip pain is likely caused by the interaction of multiple factors it will, in future research, need a multifactorial approach to not only reduce the pain but eradicate it in the majority of patients undergoing laparoscopy.

In our study, no differences were observed regarding the temperature changes during surgery which is in line with findings in other studies [8]. However, it cannot be excluded that there would have been a difference if the temperature was measured at multiple points during surgery. As temperature change was not the main clinical endpoint, we did not pay attention to a more stringent protocol to measure the temperature which could be an aim for further studies.

No significant differences were found in the length of recovery room stay and length of hospital stay. These parameters were not adequately reflected by the study design as both parameters in our hospital were administrational issues only distantly related to the actual condition of the patients. We were not aware of the influence of this fact on the results prior to the study, and it is reasonable to assume that had the differences in pain levels and morphine consumption been used in an enhanced recovery and early discharge setting, then patients in the treatment group would have been discharged earlier, on average, than the control group. It is also reasonable to assume that this would lead to substantive cost savings to the institution in the medium-term, in terms of financial costs no longer incurred. The length of recovery room stay reflects the postoperative condition of the patients better than the length of hospital stay but it was too often influenced by the time the recovery room nurses needed to prepare the patients for the general ward depending on their capacity or personal situation, as well as the time the nurses in the ward had available to move patients from the recovery room. This was a secondary endpoint and it does not relate to the actual readiness of patients to be released from the recovery room as determined by an “Apgar” type scale. As an example, the 10-point surgical Apgar score proposed by Gawande et al. based on blood loss, heart rate, and mean arterial pressure could be applied in further studies as an aid to determine patients' readiness to move out of the recovery room [27]. In our hospital, the length of hospital stay in a normal postoperative situation is quite similar for all patients after a coded-DRG surgery. This means that patients undergoing LAVH stay in the hospital irrespective of whether or not they could have been discharged earlier, and only complicated cases tend to stay longer. Therefore, in our study this parameter does not reflect patients' condition after operation. The fact that there were no significant differences in length of time in recovery room and duration of inpatient stay should thus be seen in the context that the study design did not specifically cater to the evaluation of the “fitness” of patients to be discharged, as there were other factors involved such as a standard time for a procedure. An evaluation based on the fitness of the patient to qualify for discharge would probably (based on the significant differences in the pain felt by the two treatment groups) have shown that the humidified group qualified for earlier discharge. This would give the information required, irrespective of whether or not the patient is actually discharged. In further studies conducted in hospitals with similar discharge policies, we recommend taking this aspect into account during the development of the study protocol.

### 4.3. Comparison with Relevant Findings from Other Published Studies

The results of studies conducted to investigate the influence of warm, humidified CO_2_ gas on short-term intra- and postoperative outcomes are contradictory making it difficult for clinicians to decide whether a humidification system should be implemented in the surgical routine. A recently published large-scaled RCT investigating the impact of surgical humidifier during laparoscopic appendectomy in 190 children found no statistically significant clinical benefit on postoperative pain. Furthermore, the surgical humidifier had no effect on intraoperative core temperature and postoperative recovery parameters [28]. Additionally, several meta-analyses about this topic already exist with conflicting results. Sammour et al. (2008) included 7 studies in their meta-analysis and concluded that heated and humidified insufflation gas reduces postoperative pain [29]. Another meta-analysis comprising 10 studies also revealed the reduction of postoperative pain by using humidified, heated insufflation gas [30]. This stands in contrast to a Cochrane meta-analysis published in 2011 comprising 16 studies, demonstrating that humidified and heated gas has no impact on postoperative pain or analgesic requirements overall as well as on temperature variations during surgery [8]. The meta-analysis included both gynaecological studies and studies investigating visceral surgery. Most of the studies considerably differ from each other in terms of their study design and many of the studies included small sample sizes. However, five of the 16 included studies revealed a benefit with the use of heated insufflation gas but consequently 11 studies failed to show any difference. None of the studies found that humidified and heated gas is inferior to cold-dry gas in terms of postoperative pain or analgesic requirements. As the majority of studies imply that humidified gas has no impact on postoperative outcomes, we were sceptical if we would find a significant difference between the study groups. The present study was conducted during the implementation process of surgical humidification in our department, which has a special interest and expertise in adhesion prevention [31–35]. Our initial appraisal, based on the available literature, was that humidification of the laparoscopic gas could have favourable effects on the peritoneum, especially in longer laparoscopic surgeries. The present study was aimed at investigating the extent and occurrence of short-term benefits. Therefore, it was all the more surprising that the present study revealed the differences that it did, such as the significant differences in shoulder-tip pain and postoperative morphine consumption.

As far as we know, 6 studies have so far been conducted in the field of gynaecology [1, 3, 36–39]. These studies differ from each other in terms of their study design and have also yielded variable results. In the present study, several processes were standardized for more effective comparison of the study subjects including the anesthesia protocol, the temperature measurement, and administration of pain medication. Additionally, only senior consultant physicians performed the surgery. A considerable impact on the results caused by different surgeons is not expected since LAVH is a frequently performed and relatively simple procedure in our department at a rate of 400–600 per year that is conducted in the same sequence every time and can thus be well standardized. Furthermore, subjects were selected in an unbiased fashion. It can thus be reasonably assumed that in the present study the influence of confounding factors was minimised and the observed differences in shoulder-tip pain and morphine consumption between the two groups are due to the different conditioning of the insufflated CO_2_.

### 4.4. Limitations of the Present Study

A limitation of the present study was firstly that only females were operated on and only one operation was investigated. Though the generalizability may be limited by that fact that the restrictive study design may contribute to the reason why, unlike in other studies, a difference between groups was found. However, since the main reason for shoulder-tip pain is the laparoscopy itself and not the type of surgery (except for surgery affecting the diaphragm) it is likely that the results can be generalized to other laparoscopic procedures as well. Secondly, only short-term laparoscopy was investigated and we cannot state with certainty if the effect is also present after longer laparoscopic procedures, but it is likely that there is no difference or that the effect is even greater as more damage to the peritoneal cells may occur when the pneumoperitoneum is maintained over a longer period of time. Thirdly, all patients had a vaginal tamponade until the morning of the first postoperative day and all patients had a drainage which was removed during the first postoperative day. Both may have influenced pain sensation. Since all patients received a tamponade and drainage it would not have caused a difference in pain sensation between groups. Furthermore, every patient was encouraged during every questioning to discriminate between intrapelvic pain and pain due to the vaginal tamponade and the patients confirmed that they were able to distinguish between intravaginal pressure of the tamponade and the actual intra-abdominal pain location. Finally, the number of patients investigated was not large, but according to the preceding power analysis it was adequate to answer the research question.

### 4.5. Clinical and Research Implications

The results of the study are important for clinicians as they show that with humidification of the insufflation gas, shoulder-tip pain can be significantly reduced which in turn could lead to faster recovery and earlier discharge of the patients. The cost savings through reduction of morphine usage are negligible as morphine is not expensive and this cannot be weighed against the higher additional costs of the humidification system. Nevertheless, the possible faster recovery of patients may be an important factor to consider when thinking about costs as many hospitals already strive to discharge their patients as soon as they feel ready to leave. The cost savings, in terms of costs no longer incurred, are likely to be substantive and pivotal in the short-term future as patient medical costs and reimbursement are increasingly scrutinized. Further studies therefore could also investigate the cost effectiveness of the humidification. Additionally, it could be investigated if the observed effect is even greater after longer laparoscopies but it is advisable to concentrate on well standardized procedures.

## Figures and Tables

**Table 1:** Baseline characteristics.

	Intervention group (*n* = 48)	Control group (*n* = 49)
Age	47.0 ± 8.2	46.7 ± 7.0

BMI (kg/m²)	28.9 ± 5.8	25.6 ± 3.7

Diagnosis	Uterus myomatosus: 42 (87.5)	Uterus myomatosus: 39 (79.6)
	Hyperplasia: 2 (4.2)	Hyperplasia: 4 (8.2)
	Therapy-resistant bleeding disorder: 1 (2.1)	Therapy-resistant bleeding disorder: 4 (8.2)
	Cervical carcinoma in situ: 3 (6.3)	Cervical carcinoma in situ: 2 (4.1)

Vaginal delivery (number)	0: 11 (22.9)	0: 13 (26.5)
	1: 14 (29.2)	1: 12 (24.5)
	2: 14 (29.2)	2: 21 (42.9)
	3: 6 (12.5)	3: 3 (6.1)
	4: 1 (2.1)	
	5: 2 (4.2)	

Caesarean section (number)	0: 35 (72.9)	0: 40 (81.6)
	1: 10 (20.8)	1: 6 (12.2)
	3: 3 (6.3)	2: 3 (6.1)

Diabetes	Yes: 1 (2.1)	Yes: 2 (4.1)
	No: 47 (97.9)	No: 47 (95.9)

Cigarettes per day (number)	4.8 ± 7.9	6.1 ± 9.8

Previous laparotomy	Yes: 2 (4.2)	Yes: 9 (18.4)
	No: 46 (95.8)	No: 40 (81.6)

Previous laparoscopy	Yes: 18 (37.5)	Yes: 12 (24.5)
	No: 30 (62.5)	No: 37 (75.5)

Operation time (min)	85.7 ± 28.7	82.5 ± 23.2

Length of pneumoperitoneum^*^ (min)	35.7 ± 13.8	35.0 ± 16.4

Room temperature (°C)	22.3 ± 1.1	22.3 ± 0.9

Total amount of gas (L)	34.2 ± 21.4	32.2 ± 19.1

Blood loss (mL)	97.2 ± 104.2	72.2 ± 79.9

Uterine weight (g)	214.9 ± 173.2	177.3 ± 127.8

Morphine intraoperative (mg)	0.3 ± 1.5	0.3 ± 1.1

Morphine recovery room (mg)	5.2 ± 5.2	4.5 ± 3.9

PCA-pump connection-midnight^#^ (hours)	9.8 ± 2.2	10.0 ± 2.4

Values are given as mean ± standard deviation or number (percent).

^*^summarizes the time of first and second laparoscopy.

^#^Time from connection of the PCA pump until midnight on operation day.

**Table 2:** Results.

	Intervention group	Control group	Difference between medians	Significance
			(95% confidence interval)	
Rest pain	2 h: 5.5 (0–9.3)	2 h: 4.6 (0–9.5)	2 h: −0.3 (−1.4–0.9)	2 h: *p* = 0.592
	4 h: 2.7 (0–8.6)	4 h: 3.2 (0–8.3)	4 h: −0.1 (−0.9–0.8)	4 h: *p* = 0.800
	6 h: 0.8 (0–8.9)	6 h: 1.45 (0–7)	6 h: 0.2 (−0.4–0.7)	6 h: *p* = 0.499
	24 h: 0.75 (0–6.3)	24 h: 0.6 (0–7.6)	24 h: −0.1 (−0.6–0.2)	24 h: *p* = 0.487
	48 h: 0 (0–2.5)	48 h: 0 (0–4.5)	48 h: 0 (0)	48 h: *p* = 0.641
	Total: **10.2 (0–31.7)**	Total: **11.1 (1.4–28.0)**	Total: **0.05 (−2.8–3.1)**	Total: **p** ** = 0.977**

Movement pain	2 h: 4.9 (0–9.6)	2 h: 4.6 (0–9.8)	2 h: −0.3 (−1.4–0.9)	2 h: *p* = 0.608
	4 h: 3.7 (0–8.6)	4 h: 3.8 (0–8.7)	4 h: −0.2 (−1.2–0.9)	4 h: *p* = 0.779
	6 h: 2.2 (0–8.9)	6 h: 2.3 (0–8.8)	6 h: −0.1 (−0.9–0.6)	6 h: *p* = 0.749
	24 h: 2.35 (0–8.4)	24 h: 2.5 (0–8.4)	24 h: 0.2 (−0.5–1.3)	24 h: *p* = 0.539
	48 h: 0.3 (0–5.1)	48 h: 0.5 (0–5.1)	48 h: 0 (−0.1–0.3)	48 h: *p* = 0.562
	Total:** 14.2 (0.5–28.3)**	Total:** 13.65 (0.4–33.7)**	Total:** 0.7 (−3.1–4.2)**	Total: **p** ** = 0.719**

Shoulder-tip pain	2 h: 0 (0–2.7)	2 h: 0 (0–8.7)	2 h: 0 (0)	2 h: *p* = 0.278
	Mean: 0.13	Mean: 0.65		
	4 h: 0 (0–5.4)	4 h: 0 (0–3.6)	4 h: 0 (0)	4 h: *p* = 0.235
	Mean: 0.21	Mean: 0.23		
	6 h: 0 (0–2.4)	6 h: 0 (0–7.2)	6 h: 0 (0)	6 h:
	Mean: 0.09	Mean: 0.45		Upper side *p* = 0.008
				Two-sided *p* = 0.016
	24 h: 0 (0–8.3)	24 h: 0 (0–10)	24 h: 0 (0)	24 h: *p* = 0.538
	Mean: 1.24	Mean: 1.61		
	48 h: 0 (0–8.4)	48 h: 0.1 (0–10)	48 h: 0 (0–0.4)	48 h: *p* = 0.155
	Mean: 1.23	Mean: 1.62		
	Total:	Total:	Total:	Total:
	**0.35 (0–11.2)**	**1.6 (0–24.4)**	**0 (0–2.1)**	Upper side **p** ** = 0.037**
	Mean: 2.62	Mean: 4.73		Two-sided **p** ** = 0.074**

Cough pain	2 h: 4.1 (0–9.3)	2 h: 5.2 (0–9.7)	2 h: 0.2 (−0.9–1.5)	2 h: *p* = 0.634
	4 h: 3.5 (0–8.9)	4 h: 3.0 (0–9.4)	4 h: −0.4 (−1.5–0.7)	4 h: *p* = 0.489
	6 h: 2.4 (0–8.4)	6 h: 2.65 (0–9.0)	6 h: −0.2 (−1.1–0.8)	6 h: *p* = 0.686
	24 h: 2.6 (0–8.8)	24 h: 2.5 (0–9.3)	24 h: 0 (−0.9–1)	24 h: *p* = 0.945
	48 h: 0.4 (0–8.0)	48 h: 0.5 (0–6.9)	48 h: 0 (−0.3–0.3)	48 h: *p* = 0.790
	Total:** 16 (1.9–29.8)**	Total:** 14.65 (1.2–35.1)**	Total:** −0.2 (−4–3.1)**	Total: **p** ** = 0.880**

Temperature change^*^	−0.1 (−0.7–0.7)	−0.1 (−0.7–0.5)		*p* = 0.768

Recovery room time	130 (70–1440)	135 (60–135)	0 (−20–20)	*p* = 0.994

Length of stay	6 (3–9)	6 (5–9)	0 (0-0)	*p* = 0.392

Morphine	10.5 (3.0–45.0)	13.5 (0–37.5)	3 (0–6)	Upper side *p* = 0.027
on operation day	Mean: 12.51	Mean: 14.84		Two-sided *p* = 0.054

Morphine	7.5 (0–46.5)	9 (0–36.0)	3 (0–4.5)	Upper side *p* = 0.030
24 h postoperatively	Mean: 8.59	Mean: 11.36		Two-sided *p* = 0.061

Morphine	0 (0–12.0)	0 (0–7.5)	0 (0)	*p* = 0.896
48 h postoperatively	Mean: 0.52	Mean: 0.43		

Total morphine consumption	16.5 (3.0–84.0)	24 (0–75)	6 (0–10.5)	Upper side *p* = 0.0127
	Mean: 21.65	Mean: 26.62		Two-sided *p* = 0.0253

Bolus rejected	2 (0–53)	2 (0–31)	0 (−1–1)	*p* = 0.574
on operation day	Mean: 5.02	Mean: 4.33		

Bolus rejected	0 (0–16)	0 (0–13)	0 (0)	Upper side *p* = 0.016
24 h postoperatively	Mean: 0.66	Mean: 1.96		Two-sided *p* = 0.032

Bolus rejected	0 (0-1)	0 (0-1)	0 (0)	*p* = 0.523
48 h postoperatively	Mean: 0.04	Mean: 0.02		

Total bolus rejected	**2 (0–55)**	**3 (0–37)**	**1 (0–2)**	**p** ** = 0.293**
	Mean: 5.48	Mean: 6.31		

Values are given as median (range) or mean as stated.

*p* = two-sided *p* if not otherwise stated; upper side *p* = H1: *x* tends to be greater than *y*; two-sided *p* = H1: *x* tends to be distributed differently to *y*; *x* = control group.

^*^ANOVA.
